# Bone Marrow Culture-Derived Conditioned Medium Recovers Endothelial Function of Vascular Grafts following *In Vitro* Ischemia/Reperfusion Injury in Diabetic Rats

**DOI:** 10.1155/2022/7019088

**Published:** 2022-10-14

**Authors:** Sevil Korkmaz-Icöz, Gianluca Sistori, Sivakkanan Loganathan, Alex Ali Sayour, Paige Brlecic, Tamás Radovits, Maik Brune, Matthias Karck, Gábor Szabó

**Affiliations:** ^1^Department of Cardiac Surgery, University Hospital Heidelberg, 69120 Heidelberg, Germany; ^2^Department of Cardiac Surgery, University Hospital Halle (Saale), 06120 Halle, Germany; ^3^Heart and Vascular Center, Semmelweis University, 1122 Budapest, Hungary; ^4^Department of Medicine I and Clinical Chemistry, University Hospital Heidelberg, 69120 Heidelberg, Germany

## Abstract

Ischemia/reperfusion injury (IRI) remains a challenge in coronary artery bypass grafting (CABG). Diabetic patients with coronary artery disease are more likely to require CABG and therefore run a high risk for cardiovascular complications. Conditioned medium (CM) from bone marrow-derived mesenchymal stem cells has been shown to have beneficial effects against IRI. We hypothesized that adding CM to physiological saline protects vascular grafts from IRI in diabetic rats. Bone-marrow derived cells were isolated from nondiabetic rat femurs/tibias, and CM was generated. As we previously reported, CM contains 23 factors involved in inflammation, oxidative stress, and apoptosis. DM was induced by streptozotocin administration. Eight weeks later, to measure vascular function, aortic rings were isolated and mounted in organ bath chambers (DM group) or stored in 4°C saline, supplemented either with a vehicle (DM-IR group) or CM (DM-IR+CM group). Although DM was associated with structural changes compared to controls, there were no functional alterations. However, compared to the DM group, in the DM-IR aortas, impaired maximum endothelium-dependent vasorelaxation in response to acetylcholine (DM 86.7 ± 0.1% vs. DM-IR 42.5 ± 2.5% vs. DM-IR+CM 61.9 ± 2.0%, *p* < 0.05) was improved, caspase-3, caspase-8, caspase-9, and caspase-12 immunoreactivity was decreased, and DNA strand breakage, detected by the TUNEL assay, was reduced by CM. We present the experimental finding that the preservation of vascular grafts with CM prevents endothelial dysfunction after IRI in diabetic rats. Targeting apoptosis by CM may contribute to its protective effect.

## 1. Introduction

For a large number of patients with coronary heart disease, coronary artery bypass grafting (CABG) with autologous conduits remains the most common strategy for coronary revascularization [1]. However, ischemia/reperfusion (IR) injury is an unavoidable process in cardiac surgery. Damage to the endothelium during harvest and storage (prior to anastomosis) has been identified as the main trigger of graft disease. Additionally, reperfusion itself can exacerbate damage to the ischemic tissue. While ischemic injury is mainly due to oxygen-deprived cell death, reperfusion produces inflammatory responses and generates reactive oxygen species (ROS), leading to an enhancement of apoptosis [2].

Diabetes mellitus (DM), a chronic metabolic disorder characterized by inappropriate hyperglycemia due to lack of, or resistance to insulin, is strongly associated with both microvascular and macrovascular complications [3]. Coronary artery disease is common in diabetics [4]; therefore, diabetic patients represent an important subset of patients undergoing CABG. It is well documented that DM-induced alterations in the vasculature include structural and functional changes [5], and the ability of the vascular endothelium to induce vasodilation is initially impaired. Thus, preexistent vascular damage due to DM may be aggravated by hypothermic preservation followed by warm reperfusion during CABG, and these patients present worse long-term survival rates after a CABG than nondiabetic individuals [6]. Standard intraoperative solutions (i.e., saline or blood) do not provide sufficient protection of grafts' endothelium. As a consequence, optimal storage conditions and/or specific storage solutions during surgery is crucial for CABG success.

Bone marrow-derived mesenchymal stem cells (MSCs), self-renewing and multipotent, have the potential to engraft into injured tissue where they have displayed immunomodulatory, anti-inflammatory, and tissue repair properties in preclinical and ongoing clinical trials [7, 8]. Furthermore, their capacity to differentiate into cardiomyocytes, endothelial cells, and vascular smooth muscle cells, or cell-to-cell contact was originally proposed as the principal mechanism of functional improvement. The magnitude of newly differentiated cells after MSCs transplantation is low [9]. However, there is growing evidence supporting paracrine mechanisms mediated by bioactive factors synthesized and secreted by MSCs [10, 11]. These include antiapoptotic factors (such as vascular endothelial growth factor and monocyte chemoattractant protein-1), chemokines and cytokines [12], which may contribute to the inhibition of cell death under oxidative stress, thereby inducing protection [10, 11]. Timmers et al. reported that treatment with conditioned medium (CM) from bone marrow-derived MSCs provides protection without the use of cells by reducing apoptosis and oxidative stress following myocardial IR injury [13]. Our recent studies provide evidence that preservation of brain-dead [14] or 15-month-old [15] rats' vascular grafts with CM alleviates endothelial dysfunction following IR injury. The novelty of the present study lies in the fact that CM has not been investigated in an experimental model of diabetes-associated vascular dysfunction in IR injury.

Based on the aforementioned studies, we hypothesized that bone-marrow culture-derived CM added to physiological saline protects vascular grafts from IR injury in diabetic rats. Furthermore, because of the importance of cell death, including apoptosis, following IR injury, and the identification of antiapoptotic factors in CM, we aimed to explore the possible mechanism behind CM against vascular IR injury.

## 2. Materials and Methods

### 2.1. Animals

Male Sprague-Dawley rats (Janvier Labs, Saint Berthevin, France) were housed in temperature and light-controlled rooms (22 ± 2°C with 12-12 h light-dark cycles) with food and water access ad libitum and were acclimatized for 1 week. All animals received humane care in compliance with the “Principles of Laboratory Animal Care,” formulated by the National Society for Medical Research, and with the “Guide for the Care and Use of Laboratory Animals,” prepared by the Institute of Laboratory Animal Resources and published by the National Institutes of Health (NIH Publication, 8^th^ Edition, 2011) with prior approval by the regional authorities in Karlsruhe, Germany (G122/11).

### 2.2. Induction of Diabetes Mellitus

As previously reported [16], type-1 DM was induced in rats with a single intraperitoneal dose of streptozotocin (60 mg/kg, freshly dissolved in 0.1 M citrate buffer). Seventy-two hours after streptozotocin injection, a drop of blood was collected from the tail vein, and the blood glucose concentration was determined using a digital blood glucose meter and test strips (Accu-Chek Sensor, Roche, Mannheim, Germany). Animals with a random blood glucose level of >15 mmol/L were considered diabetic and were included in the study (DM group). Control animals received only the vehicle buffer.

### 2.3. Preparation of Bone Marrow Derived MSC-CM

As previously reported [14], CM was prepared from bone marrow-derived cells isolated from nondiabetic young rats (8-12 weeks old). Briefly, the animals were euthanized with an overdose of sodium pentobarbital (100 mg/kg, intraperitoneally). Bone marrow was collected by flushing femurs and tibias with Dulbecco's phosphate-buffered saline (Sigma, St. Louis, MO, USA). The cells were suspended in MSC Expansion Medium (R&D System, Minneapolis, MN, USA) and then incubated at 37°C with 5% CO_2_. The medium containing nonadherent cells was discarded 24 h later, and fresh MSC Expansion Medium was added. After isolation, the medium was changed every 3 days, and the primary cells were subcultured 1 : 3 at 80% confluence. MSC-CM was obtained at Passage 3. After the MSC cells had reached greater than 80% confluence, 15 mL medium was aspirated, and the cells were rinsed 3 times with Dulbecco's phosphate-buffered saline. Then, 7 mL Dulbecco's modified Eagle's medium (D-MEM) (Life Technologies, Grand Island, NY, USA) was added to the culture dishes with cells, and the culture dishes were incubated for 24 h. Primary CM was collected and centrifuged by ultrafiltration units (Amicon® Ultra-15 Centrifugal Filter Units, Merck Millipore Ltd. Tullagreen, Carrigtwohill, Co Cork IRL) at 4,500 g for 4 h at 4°C to increase protein concentration (i.e. to yield concentrated CM). The protein concentration of the CM was quantified with Bradford protein assay to ensure that a final concentration of 0.5 mg/mL of CM was used. Equal volume of D-MEM was used as a vehicle (nonconditioned medium).

### 2.4. In Vitro Model of Vascular Dysfunction Induced by Cold Ischemic Storage and Warm Reperfusion

#### 2.4.1. Preparation of Aortic Rings

As previously reported [14], rats were anesthetized with sodium pentobarbital (60 mg/kg, intraperitoneally), blood was collected from the abdominal vena cava, and subsequently, animals were euthanized by bleeding out. The descending thoracic aorta was explanted. Periadventitial fat and connective tissues around the aorta were carefully cleaned under magnification in either cold (+4°C) Krebs-Henseleit-solution (KHL) containing 118 mM NaCl, 4.7 mM KCl, 1.2 mM KH_2_PO_4_, 1.2 mM MgSO_4_, 1.77 mM CaCl_2_, 25 mM NaHCO_3_, and 11.4 mM glucose (pH = 7.4) or cold physiological saline solution. The aorta was subsequently cut into 4 mm wide rings. KHL solution is used during the organ bath experiments. The rings of control and DM groups were immediately mounted in organ bath chambers; therefore, they were prepared in KHL solution. However, the model of IR in our study calls for an overnight cold ischemic phase in cold NaCl 0.9%. Therefore, the aortic rings of all IR groups were prepared in saline. After this cold incubation, these rings were also mounted in organ bath chambers filled with KHL solution.

#### 2.4.2. Conservation of Aortic Rings and Experimental Groups

As previously reported [17], the thoracic aortic rings were placed in closed, air-free tubes filled with saline-supplemented with either D-MEM vehicle [control-IR (*n* = 27-31 rings, 7-8 rats) and DM-IR (*n* = 40-44 rings, 10-11 rats) groups] or CM [control-IR+CM (*n* = 36-40 rings, 9-10 rats) and DM-IR+CM (*n* = 43-44 rings, 11 rats) groups] and stored for 24 h at 4°C. The tubes had previously been equilibrated with nitrogen to extrude oxygen from the solution. After cold ischemic conservation, the rings were mounted in organ bath chambers. To simulate free radical burst and endothelial dysfunction, which usually occurs during reperfusion *in vivo*, hypochlorite was added to the baths (200 *μ*M, 30 min). Aortic rings in the control (*n* = 31-32 rings, 8 rats) and DM (*n* = 31-32 rings, 8 rats) normoxia groups did not undergo cold ischemic storage and hypochlorite incubation but were immediately mounted in organ baths.

### 2.5. *Ex Vivo* Vasoreactivity Assessment

As previously reported [18], the aortic rings were mounted on stainless steel hooks and subjected to a passive tension of 2 g in organ baths (Radnoti Glass Technology, Monrovia, CA, USA), containing 30 mL of KHL and continuously gassed with 95% O_2_-5% CO_2_ at 37°C. The tissue was equilibrated for 60 minutes with a change of KHL every 30 minutes as a precaution against interfering metabolites. During this period, the tension was periodically adjusted to 2 grams. At the beginning of each experiment, potassium chloride (KCl, 80 mM) was used to test viability, prepare the vessel rings for stable contractions, and establish reproducible dose-response curves to other vasoactive agents. This was maintained for approximately 30 minutes, after which aortic rings were washed until resting tension was again obtained. An *α*-adrenergic receptor agonist, phenylephrine (PE, 10^−9^-10^−5^ M), was used to precontract the rings until a stable plateau was reached. Relaxation responses were examined by adding cumulative concentrations of an endothelium-dependent vasorelaxant, acetylcholine (ACh, 10^−9^-10^−4^ M), and an endothelium-independent dilator, sodium nitroprusside (SNP, 10^−10^-10^−5^ M). Relaxation is expressed as the percentage of contraction induced by PE in grams. Contractility in response to PE is expressed as the percentage of KCl response in grams. The half-maximal effective concentration (EC_50_) was determined from each individual concentration-response curve to PE, ACh, or SNP by sigmoidal fits using Origin 7.0 (Microcal Software, Northampton, Massachusetts, USA). The sensitivity pD_2_ (-logEC_50_) was then calculated.

### 2.6. Aortic Histomorphometry

As previously reported [19], distal regions of the aortic segments were fixed in a buffered paraformaldehyde solution (4%) and embedded in paraffin. Then, 5 *μ*m thick sections were placed on adhesive slides and stained with hematoxylin and eosin as described elsewhere [18]. The aortic morphometric measurements, including lumen cross-sectional area, intima-media area, and intima-media width, were determined under a microscope using Cell^A software (Olympus Soft Imaging Solutions GmbH, Germany), then normalized to body weight [20]. Additionally, the intima-media/lumen area ratio was calculated. The evaluation was conducted by an analyst blinded to the experimental groups.

### 2.7. Immunohistochemical Staining for Caspase-3, Caspase-8, Caspase-9, and Caspase-12

Hydrogen peroxide (3%) was used to quench endogenous peroxidase activity for 30 min. Then, sections were pretreated using heat-induced antigen retrieval with sodium citrate buffer (pH = 6) for 20 min in a microwave oven at 700 Watt and 2% normal serum in buffer to block sections for 30 min at room temperature. After that, the sections were incubated with mouse monoclonal IgG anti-caspase-3 (1 : 1000; antibodies-online GmbH, Aachen, Germany), rabbit polyclonal IgG anti-caspase-8 (1 : 1000; Novus Biologicals, Littleton, Colorado, USA), mouse monoclonal IgG anti-caspase-9 (1 : 50; Santa Cruz Biotechnology, Dallas, Texas, USA), and rabbit polyclonal IgG anti-caspase-12 (1 : 200; Novus Biologicals, Littleton, Colorado, USA) antibodies for 2 h at room temperature. Then, biotinylated secondary antibody was diluted in blocking serum buffer (1 : 50), and sections were covered for 30 min at room temperature. Biotinylated secondary antibody was detected by avidin-biotinylated complex (ABC) reagent (VECTASTAIN universal elite ABC kit, Burlingame, USA). Afterwards, slides were developed with DAB (3,3-diaminobenzidine) substrate that produced a dark brown reaction product in the presence of horseradish peroxidase enzyme and was used in double labelling applications (VECTOR DAB kit, Burlingame, USA). Lastly, slides were dehydrated, cleared, and permanently mounted with ProTaqs Mount Aqua (Quartett, Berlin, Germany). Counterstaining was performed with haematoxylin. Semiquantitative immunohistochemical analysis was performed using conventional microscopy based on the intensity score multiplied by area score (0-12). The evaluation of 4-5 randomized, nonoverlapping fields per aorta per rat was performed in a blinded fashion.

### 2.8. Terminal Deoxynucleotidyl Transferase-Mediated dUTP Nick End-Labeling (TUNEL) Staining

We performed TUNEL assay to detect DNA strand breaks as previously described [21, 22]. A commercially available kit (Roche Diagnostics Indianapolis, IN, USA) was used according to the manufacturer's manual. Sections were deparaffinized with xylene and passaged through decreasing concentrations of ethanol, washed with PBS, and digested by diluted DNase-free Proteinase-K (1 : 10, Millipore, 21627) to retrieve antigenic epitopes for 20 min, RT. Then, sections were washed with phosphate buffer saline (PBS, 1X) for 2x5 min. After blocking endogenous peroxidases with 3% hydrogen peroxide for 5 min in the dark, sections were washed in PBS 2 × 5 min. Positive and negative controls were prepared by adding 50 *μ*L DNase I for positive or 50 *μ*L of pure labeling reagent for negative controls followed by 5 min washing in PBS. Then, sections were incubated with TUNEL reaction mixture (Roche Diagnostics, Mannheim, Germany) in a dark, humidified atmosphere for 60 min at 37°C. The sections were then washed with PBS for 3 × 5 min. The slides were mounted using 4′,6-diamidino-2-phenylindole- (DAPI-) Fluoromount-GTM (SouthernBiotech, Birmingham, USA), covered with cover glass, and analyzed under a fluorescence microscope. The number of TUNEL-positive endothelial and smooth muscle cells was expressed as the ratio of 4′,6-diamidino-2-phenylindole- (DAPI-) TUNEL double-labeled nuclei to the total number of nuclei stained with DAPI. The evaluation of 4 randomized nonoverlapping fields per aorta per rat was completed in a blinded fashion.

### 2.9. Statistical Analysis

All data are expressed as the mean ± standard error of the mean (SEM). Statistical analyses were performed using GraphPad Prism 7.02 software (GraphPad Software, Inc., CA, USA). The Shapiro-Wilk normality test was used to assess normal distribution before statistical tests were applied. For data with normal distribution, two-sample Student's *t*-test was used to analyze the differences between the control and diabetic groups. If data were not normally distributed, a nonparametric Mann–Whitney *U* test was applied. In all other cases, one-way ANOVA and Tukey's post hoc test were carried out for multiple comparisons. If the data were not normally distributed, the nonparametric Kruskal-Wallis test followed by Dunn's post hoc test were used. The value of *p* < 0.05 was regarded as statistically significant.

## 3. Results

### 3.1. Characterization of the Diabetic Rat Model and Effect of IR Injury in the Diabetic Rat Aorta

#### 3.1.1. Blood Glucose

Eight weeks after the injection of streptozotocin (and confirmation of diabetes), blood glucose concentrations were significantly increased in DM rats compared to controls (29.4 ± 1.4 versus 6.8 ± 0.4 mmol/L, *p* < 0.05).

#### 3.1.2. Aortic Morphometry

Diabetic rats had significantly lower body weight compared to controls (Table 1(a)). Morphometrical analyses of aortas revealed that intima-media wall thickness, intima-media wall cross-section area, and lumen area normalized to body weight were significantly higher, while the wall : lumen area ratio was lower in diabetic rats compared to the control group (Table 1(a); see Supplementary Online Figure S1).

#### 3.1.3. Contractile Responses in the Aortic Rings

**Figure 1:**
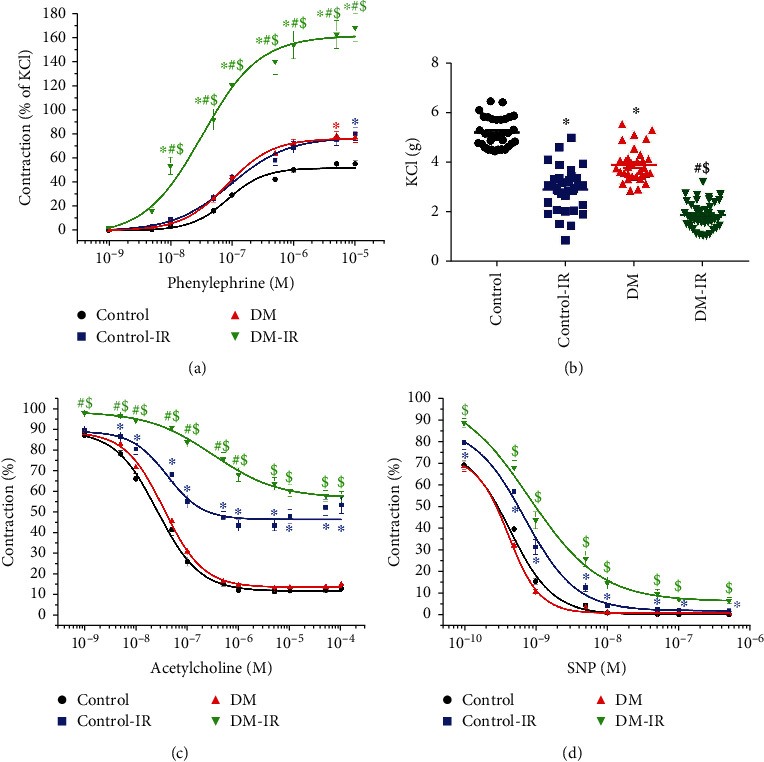
Effect of ischemia/reperfusion (IR) injury on contraction and relaxation in the aorta of nondiabetic control and diabetic rats. Contractile responses for (a) phenylephrine [expressed as the percentage of the maximum contraction induced by potassium chloride (KCl)] and (b) high potassium K^+^-induced depolarization (control, *n* = 32 rings from 8 rats; control-IR, 31 rings form 8 rats; DM, 32 rings from 8 rats; DM-IR, 40 rats from 10 rats) and (c) acetylcholine-induced endothelium-dependent vasorelaxation (control, *n* = 31 rings from 8 rats; control-IR, 31 rings form 8 rats; DM, 31 rings from 8 rats; DM-IR, 44 rats from 11 rats) and (d) sodium nitroprusside- (SNP-) induced endothelium-independent vasorelaxation (control, *n* = 32 rings from 8 rats; control-IR, 31 rings form 8 rats; DM, 32 rings from 8 rats; DM-IR, 44 rats from 11 rats) of isolated thoracic aortic rings obtained from nondiabetic and diabetic rats. DM indicates diabetes mellitus. Data are represented as the mean ± SEM. ^∗^*p* < 0.05 versus control, ^#^*p* < 0.05 versus control-IR, and ^$^*p* < 0.05 versus DM.

Exposure of aortic segments to PE (10^−9^-5 × 10^−5^ M), expressed as the percentage of 80 mM KCl-induced contraction, led to a concentration-dependent increase in tension in all experimental groups (Figure 1(a)). DM significantly increased maximum contractile responses to PE (Table 1(b), Figure 1(a)) and decreased high K^+^-induced depolarization (Table 1(b), Figure 1(b)). The contractile response to PE was significantly increased in the control-IR compared to controls, increased in the DM-IR compared to DM aortic rings, and further increased in the DM-IR compared to control-IR rats (Table 1(b), Figure 1(a)). Aortic sensitivity (pD_2_-value) to PE was significantly greater in the DM-IR group compared with both control-IR and DM (Table 1(b)). IR injury significantly decreased the vasoconstrictive responses to KCl in both control-IR and DM-IR groups compared to their respective controls (Table 1(b), Figure 1(b)). Furthermore, high K^+^-induced depolarization was further reduced in the DM-IR rings compared to the control-IR group (Table 1(b), Figure 1(b)).

#### 3.1.4. Relaxant Responses in the Aortic Rings

Figures 1(a) and 1(b) show concentration-dependent relaxation induced by 10^−9^-10^−4^ M ACh and 10^−10^-10^−5^ M SNP in aortic rings precontracted with 10^−6^ M PE, respectively. Neither endothelium-dependent vasorelaxation to ACh (Table 1(b), Figure 1(c)) nor endothelium-independent relaxation to SNP (Table 1(b), Figure 1(d)) was altered in the diabetic rats compared to the nondiabetic group. IR injury significantly decreased ACh-induced relaxation in both control-IR and DM-IR groups compared to their respective controls (Table 1(b), Figure 1(c)). Furthermore, vascular relaxation to ACh was further reduced in the DM-IR compared to the control-IR group (Figure 1(c)). The adverse impact of IR injury on *R*_max_ to ACh was significantly increased in aortic rings from diabetic animals as compared with those from nondiabetic animals, as demonstrated by normalization to controls (difference of *R*_max_ to ACh ratio : DM-IR/DM 51 ± 3% vs. control-IR/control 36 ± 3%, *p* = 0.0009). The sensitivity (pD_2_-value) of aortic rings to ACh was significantly reduced in the DM-IR compared with both control-IR and DM groups (Table 1(b)). IR injury significantly decreased SNP-induced relaxation in both the control-IR and DM-IR groups compared to their respective controls (Figure 1(d)). Furthermore, the concentration-response curve to SNP in aortas from both IR groups was shifted to the right compared with their respective controls (Table 1(b), Figure 1(d)).

#### 3.1.5. Caspase-3, Caspase-8, Caspase-9, and Caspase-12 Expression in the Aorta

**Figure 2:**
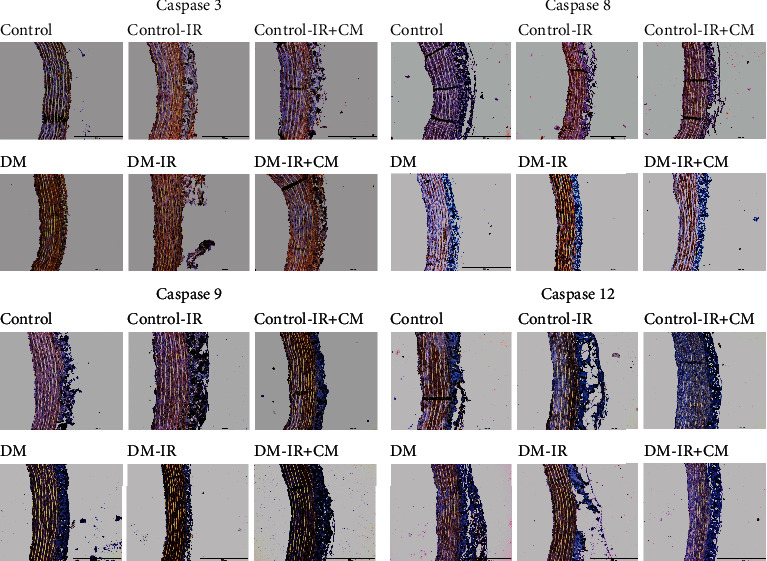
Effects of bone marrow culture-derived conditioned medium (CM) against ischemia/reperfusion (IR) injury on caspase-3, caspase-8, caspase-9, and caspase-12 expression. Representative images of caspase-3, caspase-8, caspase-9, and caspase-12 immunohistochemistry staining (brown staining, ×10 magnification, scale bar = 100 *μ*m) in the aortic rings obtained from nondiabetic control and diabetic rats. DM indicates diabetes mellitus.

**Figure 3:**
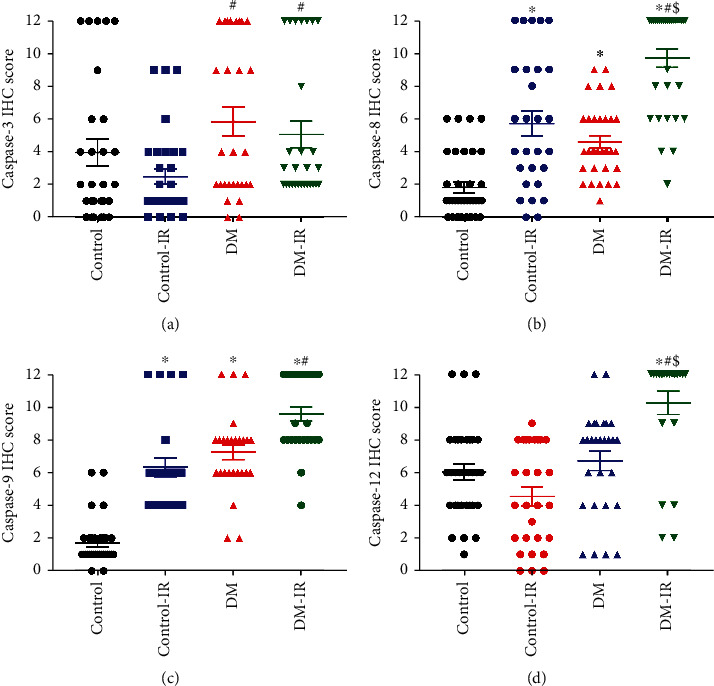
Effect of ischemia/reperfusion (IR) injury on aortic caspase-3, caspase-8, caspase-9, and caspase-12 expression in control and diabetic rats. Semiquantitative scoring of (a) caspase-3 (control, *n* = 7 rats; control-IR, *n* = 8 rats; DM, *n* = 7 rats; DM-IR, *n* = 7 rats), (b) caspase-8 (control, *n* = 8 rats; control-IR, *n* = 7 rats; DM, *n* = 8 rats; DM-IR, *n* = 8 rats), (c) caspase-9 (control, *n* = 8 rats; control-IR, *n* = 6 rats; DM, *n* = 7 rats; DM-IR, *n* = 7 rats), and (d) caspase-12 (control, *n* = 8 rats; control-IR, *n* = 7 rats; DM, *n* = 7 rats; DM-IR, *n* = 6 rats) immunohistochemical staining of thoracic aortic rings obtained from nondiabetic and diabetic rats. DM indicates diabetes mellitus. The evaluation was conducted by two examiners blinded to the experimental groups from 4 randomized nonoverlapping fields per aorta per rat. Data are represented as the mean ± SEM and correspond to the mean of all pictures. ^∗^*p* < 0.05 versus control, ^#^*p* < 0.05 versus control-IR, and ^$^*p* < 0.05 versus DM.

Figure 2 displays representative images of caspase-3, caspse-8, caspase-9, and caspase-12 immunohistochemical staining. Whereas the immunohistochemistry for caspase-3 (score : 5.9 ± 0.9 vs.4.0 ± 0.8, *p* < 0.05), caspase-8 (score : 4.6 ± 0.4 vs.1.8 ± 0.3, *p* < 0.05), and caspase-9 (score : 7.3 ± 0.5 vs.1.9 ± 0.3, *p* < 0.05) were significantly increased in the aortic wall of diabetic rats compared to controls, the expression of caspase-12 (score : 6.7 ± 0.6 versus 5.8 ± 0.6, *p* > 0.05) was similar. Furthermore, immunohistochemical analysis showed that IR injury significantly increased immunoreactivity for caspase-8 in both control-IR and DM-IR, caspase-9 in control-IR, and caspase-12 in the DM-IR groups compared to their corresponding controls (Figures 3). Additionally, the combined effect of DM and IR injury significantly further increased caspase-3, caspase-8, caspase-9, and caspase-12 immunoreactivity in the DM-IR aortic rings compared to the control-IR group (Figures 3).

#### 3.1.6. DNA Strand Breaks in the Aorta

**Figure 4:**
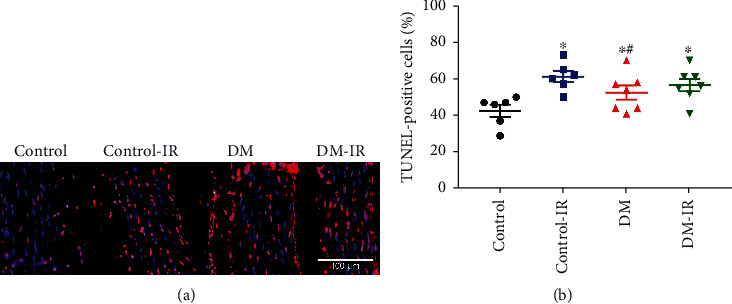
Effect of ischemia/reperfusion (IR) injury on DNA strand breaks in nondiabetic control and diabetic rats. (a) Representative merged micrographs of aortic tissue shows pink nuclei with fragmented DNA, visualized by terminal deoxynucleotidyl transferase-mediated dUTP nick end-labeling (TUNEL) staining and blue nuclei represents 4′,6-diamino-2-phenylindole staining (×40 magnification; scale bar: 100 *μ*m) followed by (b) quantification of TUNEL-positive cells. DM indicates diabetes mellitus. The evaluation was conducted by two examiners blinded to the experimental groups from 4 randomized nonoverlapping fields per aorta per rat. Data are represented as the mean ± SEM. ^∗^*p* < 0.05 versus control, ^#^*p* < 0.05 versus control-IR, ^$^*p* < 0.05 versus DM. Control, *n* = 6 ratscontrol control-IR, *n* = 7 ratscontrol DM, *n* = 7 ratscontrol DM-IR, *n* = 7 rats.

TUNEL assay was performed to detect DNA breaks formed when DNA fragmentation occurs in the last phase of apoptosis. The number of TUNEL-positive nuclei was significantly increased in the DM group compared to controls (Figure 4). Furthermore, IR injury significantly increased the number of TUNEL-positive nuclei in the control-IR compared to controls, whereas it had no effect on the inherently elevated TUNEL positivity in the DM-IR aortic rings compared to DM group (Figure 4).

### 3.2. Effect of CM against IR Injury in the Control and Diabetic Rat Aorta

#### 3.2.1. Characterization of CM by Protein Array

We previously reported the relative expression of proteins in our CM by rat cytokine antibody array coated with 90 antibodies (BioCat GmbH, Heidelberg, Germany) [14]. This array showed that CM contains 23 proteins involved in either apoptosis, inflammation, or oxidative stress (Online Table S1) [14].

#### 3.2.2. Effects of CM on Contractile Responses after IR Injury

**Figure 5:**
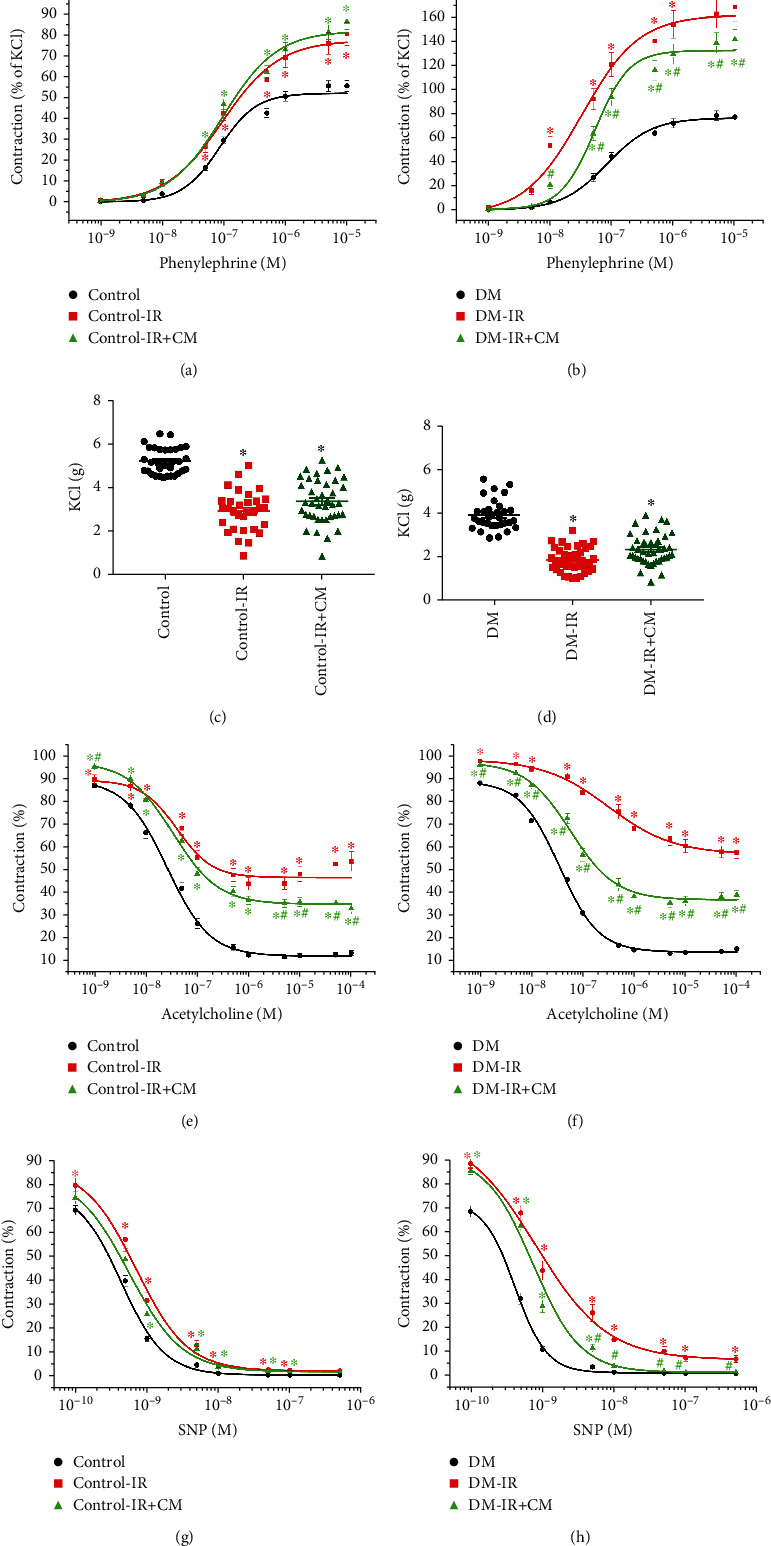
Effects of bone marrow culture-derived conditioned medium (CM) against ischemia/reperfusion (IR) injury on contraction and relaxation in the aorta of nondiabetic control and diabetic rats. Contractile responses for (a, b) phenylephrine (expressed as the percentage of 80 mM potassium chloride (KCl)-induced contraction) and (c, d) high potassium K^+^-induced depolarization (control, *n* = 32 rings from 8 rats; control-IR, *n* = 31 rings from 8 rats; control-IR+CM, *n* = 40 rings from 10 rats; DM, *n* = 32 rings from 8 rats; DM-IR, *n* = 40 rings from 10 rats; DM-IR+CM, *n* = 43 rings from 11 rats), and (e, f) acetylcholine-induced endothelium-dependent vasorelaxation (control, *n* = 31 rings from 8 rats; control-IR, *n* = 31 rings from 8 rats; control-IR+CM, *n* = 40 rings from 10 rats; DM, *n* = 31 rings from 8 rats; DM-IR, *n* = 44 rings from 11 rats; DM-IR+CM, *n* = 44 rings from 11 rats) and (g, h) sodium nitroprusside-induced endothelium-independent vasorelaxation (control, *n* = 32 rings from 8 rats; control-IR, *n* = 31 rings from 8 rats; control-IR+CM, *n* = 40 rings from 10 rats; DM, *n* = 32 rings from 8 rats; DM-IR, *n* = 44 rings from 11 rats; DM-IR+CM, *n* = 43 rings from 11 rats) of isolated thoracic aortic rings obtained from control and diabetic rats. DM indicates diabetes mellitus. Data are represented as the mean ± SEM. ^∗^*p* < 0.05 versus corresponding control or DM; ^#^*p* < 0.05 versus corresponding control-IR or DM-IR.

The increased vasoconstrictive response to PE in the DM-IR group compared to the DM group was significantly decreased by preservation of aortic rings with CM (Table 2(b), Figure 5(b)). However, CM had no effect on nondiabetic rats in these terms (Table 2(a), Figure 5(a)). Furthermore, increased sensitivity (pD_2_-value) to PE in the DM-IR group was significantly reduced by CM treatment (Table 2(b)). CM treatment had no effect on decreased maximum contractile response to high K^+^-induced depolarization in both control-IR and DM-IR groups (Table 2, Figures 5(c) and 5(d)).

#### 3.2.3. Effects of CM on Endothelium-Dependent Vasorelaxation after IR Injury

IR-induced decreased endothelium-dependent vasorelaxation in responses to ACh in both control-IR and DM-IR groups was significantly improved by preservation of aortic rings with CM (Table 2, Figures 5(e) and 5(f)). Furthermore, reduced sensitivity (pD_2_-value) to ACh was significantly improved by CM in diabetic aortic rings (Table 2(b)). In the DM group, CM resulted in increased *R*_max_ to ACh compared to nondiabetic ones, according to normalization to controls (difference of *R*_max_ to ACh ratio: diabetic-IR/diabetic 52 ± 5% vs. control-IR/control 19 ± 4%, *p* < 0.001).

#### 3.2.4. Effects of CM on Endothelium-Independent Vasorelaxation after IR Injury

Decreased endothelium-independent vasorelaxation to SNP in the DM-IR rings compared to the DM group was significantly increased by CM treatment (Table 2B, Figure 5(h)). Nevertheless, CM had no effect on impaired relaxation induced by SNP on nondiabetic rats (Table 2(a), Figure 5(g)). The preservation of aortic rings with CM had no effect on IR-induced right shift of the concentration-response curves for SNP in both control and DM groups (Table 2, Figures 5(g) and 5(h)).

#### 3.2.5. Effects of CM on Caspase-3, Caspase-8, Caspase-9, and Caspase-12 Expression after IR Injury

**Figure 6:**
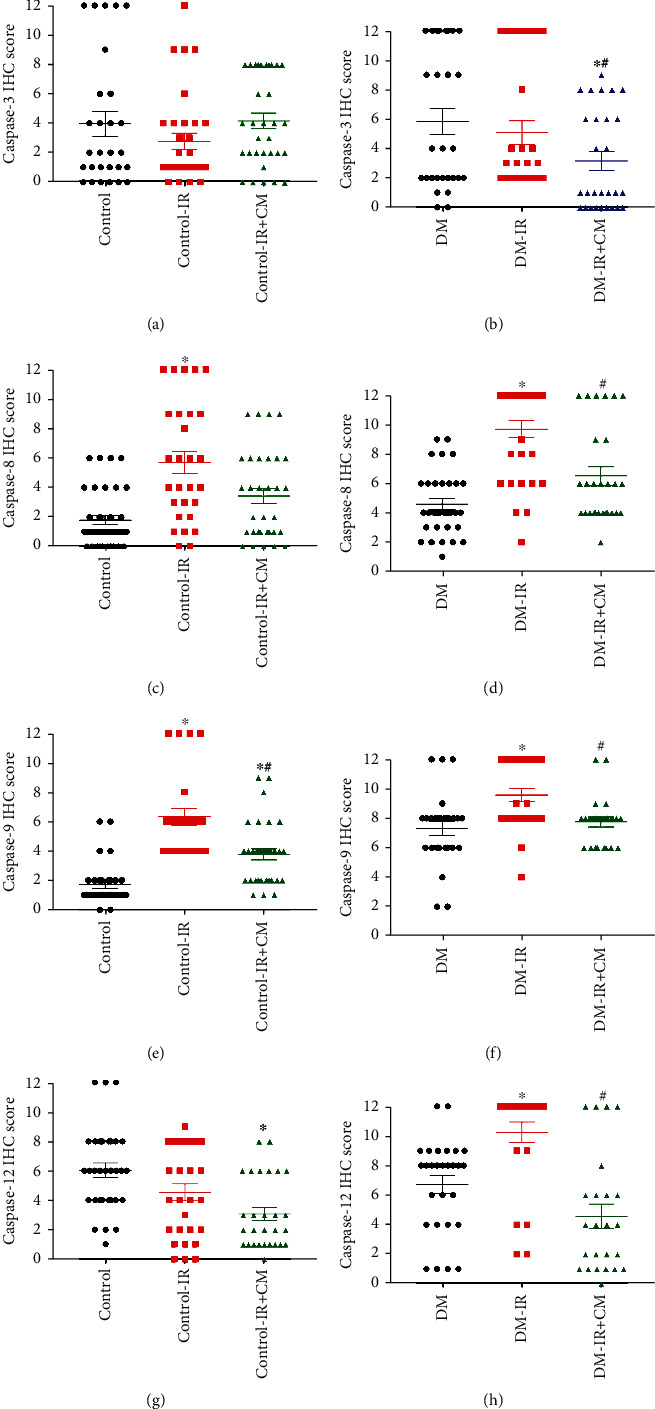
Effects of bone marrow culture-derived conditioned medium (CM) against ischemia/reperfusion (IR) injury on caspase-3 (control, *n* = 7 rats; control-IR, *n* = 8 rats; control-IR+CM, *n* = 8 rats; DM, *n* = 7 rats; DM-IR, *n* = 7 rats; DM-IR+CM, *n* = 7 rats), caspase-8 (control, *n* = 8 rats; control-IR, *n* = 7 rats; control-IR+CM, *n* = 8 rats; DM, *n* = 8 rats; DM-IR, *n* = 8 rats; DM-IR+CM, *n* = 7 rats), caspase-9 (control, *n* = 8 rats; control-IR, *n* = 6 rats; control-IR+CM, *n* = 8 rats; DM, *n* = 7 rats; DM-IR, *n* = 7 rats; DM-IR+CM, *n* = 6 rats), and caspase-12 (control, *n* = 8 rats; control-IR, *n* = 7 rats; control-IR+CM, *n* = 7 rats; DM, *n* = 7 rats; DM-IR, *n* = 6 rats; DM-IR+CM, *n* = 6 rats) expression. Semiquantitative scoring of (a, b) caspase-3, (c, d) caspase-8, (e, f) caspase-9, and (g, h) caspase-12 immunohistochemical staining in the aortic rings obtained from nondiabetic control and diabetic rats. DM indicates diabetes mellitus. The evaluation was conducted by two examiners blinded to the experimental groups from 4 randomized non-overlapping fields per aorta per rat. Data are represented as the mean ± SEM and correspond to the mean of all pictures. ^∗^*p* < 0.05 versus corresponding control or DM; ^#^*p* < 0.05 versus corresponding control-IR or DM-IR.

The preservation of DM-IR aortic rings with CM showed significantly decreased caspase-3, caspase-8, caspase-9, and caspase-12 immunoreactivity compared to the vehicle-treated DM-IR group (Figures 2 and 6). In control-IR+CM rings caspase-9 immunoreactivity was significantly decreased, while caspase-8 and caspase-12 immunoreactivity showed a tendency toward less expression without reaching statistical significance compared to the control-IR group. However, CM had no effect on caspase-3 (Figures 2 and 6).

#### 3.2.6. Effects of CM on DNA Strand Breaks after IR Injury

**Figure 7:**
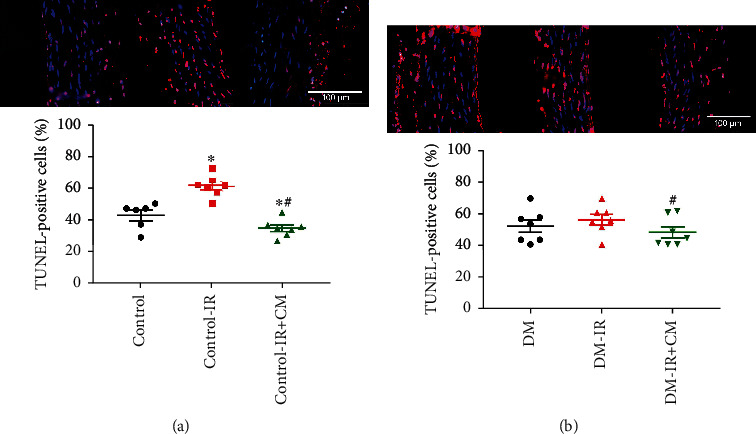
Effects of bone marrow culture-derived conditioned medium (CM) against ischemia/reperfusion (IR) injury on aortic DNA strand breaks. Representative merged micrographs of aortic tissue shows pink nuclei with fragmented DNA, visualized by terminal deoxynucleotidyl transferase-mediated dUTP nick end-labeling (TUNEL) staining and blue nuclei represents 4′,6-diamino-2-phenylindole staining (magnification ×40; scale bar: 100 *μ*m) followed by quantification of TUNEL-positive cells in the aortic rings obtained from (a) nondiabetic control and (b) diabetic rats. DM indicates diabetes mellitus. The evaluation was conducted by two examiners blinded to the experimental groups from 4 randomized non-overlapping fields per aorta per rat. Data are represented as the mean ± SEM. ^∗^*p* < 0.05 versus corresponding control or DM, ^#^*p* < 0.05 versus corresponding control-IR or DM-IR. Control, *n* = 6 rats; control-IR, *n* = 7 rats; control-IR+CM, *n* = 7 rats; DM, *n* = 7 rats; DM-IR, *n* = 7 rats; DM-IR+CM, *n* = 7 rats.

Increased numbers of TUNEL-positive cells in the control-IR rings compared to controls, was significantly decreased by CM treatment (Figure 7(a)). Additionally, we observed a significant decrease in the number of TUNEL-positive nuclei in the DM-IR+CM rings compared to the DM-IR group (Figure 7(b)).

## 4. Discussion

The present study examined bone marrow culture-derived CM's therapeutic potential against IR injury in diabetic rat vascular grafts for the first time. We presented the experimental finding that the preservation of aortic rings with CM preserves endothelial function after cold ischemic storage followed by warm reperfusion in diabetic rats. Its protective effect may be related, in part, to the prevention of caspase-mediated apoptosis.

CABG and peripheral vascular bypass procedures include the use of autologous blood vessels as a mode of revascularization [23]. However, CABG may cause further endothelial dysfunction in diabetic patients. The vasoprotective function of endothelial cells is associated with the synthesis and release of endothelium-derived relaxing factors such as nitric oxide (NO) [24, 25], prostacyclin (PGI_2_) [26], and endothelium-derived hyperpolarizing factor (EDHF) [27], as well as vasoconstrictor factors (endothelin, superoxide anion, and thromboxane) to regulate vasomotor tone. Without adequate secretion of mediators to promote vasodilation, anti-aggregation, and fibrinolysis, both DM and IR injury would contribute to vascular graft damage after CABG. Mechanisms underlying IR injury include increases in calcium and oxidative stress, excess endoplasmic reticulum stress, mitochondrial dysfunction, inflammation, and apoptosis [28]. In the present study, we have shown that IR injury further impaired vascular reactivity (contraction/relaxation) in diabetic aortas, suggesting reduced ischemic tolerance at functional levels in DM groups compared to nondiabetic ones. Moreover, IR injury enhanced caspase-3, caspase-8, caspase-9, and caspase-12-mediated apoptosis and increased DNA breaks in diabetic rats compared to controls. Clinical need demands improved graft preservation strategies to reduce injury during surgical harvest and after the reestablishment of blood flow [17]. To the best of our knowledge, this is the first study investigating the preservation of grafts in physiological saline enriched with CM in an experimental model of diabetes-associated vascular dysfunction in IR injury.

As previously mentioned, MSCs secrete molecules (e.g., chemokines, cytokines, growth factors and other supportive substances), that may contribute to graft protection in a paracrine manner under various experimental conditions [10, 11]. We recently characterized bone marrow culture-derived cell free CM, using an antibody array against 90 specified rat proteins. We identified the coexistence of 39 factors in CM [14] (*See Supplementary* Table S1). Apoptosis is considered an important mechanism of IR- and DM-induced cell dysfunction leading to tissue damage. Caspase-8, caspase-9, and caspase-12 are crucial molecules of three apoptosis pathways, namely, cytokines/Fas-mediated extrinsic pathway, mitochondrial-mediated intrinsic pathway, and endoplasmic reticulum/calcium-mediated pathway, respectively. These pathways result in caspase-3 activation and subsequently in cell death [29]. There is evidence that CM inhibits increased apoptosis [30]. However, there are no reports investigating the beneficial effect of CM against apoptosis in diabetic vascular grafts submitted to IR injury. In the present study, increased DNA breakage/nuclear fragmentation (detected by TUNEL assay) and higher caspase-8 and caspase-9 immunoreactivity after IR injury was significantly decreased by CM. This indicates that CM reduced apoptosis in vascular grafts obtained from diabetic rats. Recently, we reported that an increased number of TUNEL-positive nuclei was reduced by preservation of cardiac grafts with CM from brain-dead donor rats [31]. In line with these observations, tissue inhibitor of metalloproteinase- (TIMP-) 1, also characterized in our CM, has been demonstrated to have functions independent of blocking matrix metalloproteinase (MMP) activity. TIMP-1 inhibits programmed cell death by increasing the expression of anti-apoptotic genes [32]. It has been shown that vascular endothelial growth factor, another factor present in our CM, mediates numerous pro-survival pathways, including the inhibition of apoptosis of the vascular endothelium [33]. Evidence indicates that various stimuli, such as high glucose and oxidative stress, can lead to endothelial dysfunction, in part, through disturbance of endoplasmic reticular function [34, 35]. Therefore, we sought to evaluate the protective action CM has against IR injury-induced caspase-12 expression in vascular grafts obtained from diabetic animals. We found that increased caspase-12 immunoreactivity due to IR injury was significantly reduced by CM in diabetic rats. In accordance with these results, the administration of activin A, a protein found in the CM, has been suggested to inhibit endoplasmic reticulum stress-induced apoptotic and autophagic cell death [36]. Caspase-3, which interacts with caspase-8, caspase-9, and caspase-12, was also significantly decreased by CM in diabetic aortic rings submitted to IR injury. Furthermore, we have recently found that caspase-3, caspase-8, and caspase-12 immunoreactivity was significantly decreased after treatment with CM in vascular grafts from brain-dead rats following IR injury [14]. Taken together, CM prevents IR injury-induced impaired contractile response to the *α*-adrenergic receptor agonist PE, endothelial dysfunction and impaired vascular smooth muscle relaxation (evidenced by altered relaxation responses and sensitivities of aortic rings to ACh and SNP) through the reduction of caspase-3, caspase-8, caspase-9, and caspase-12 expression in diabetic rings. However, CM likely mobilizes additional important signaling pathways underlying its protective effect, such as oxidative stress/antioxidant pathways and inflammatory response signaling [37]. Furthermore, specific pathways involved in diabetic vascular complications were not investigated in the present study, and their potential role in CM protective effect must be considered. Even though our findings do not currently provide direct mechanistic evidence that the “cocktail” of various soluble factors secreted and identified in the CM are in fact responsible for the improved endothelial function, it does show that CM preserves endothelial dysfunction by inhibiting apoptosis. More investigations are needed to elucidate the role of these and other factors identified in CM. It should also be noted that CM contains both pro- and anti-apoptotic factors. Apoptosis is essential for the maintenance of tissue homeostasis [38]. We speculate that the balance between pro- and antiapoptotic family members in CM maintains tissue homeostasis, and that its dysregulation could result in tissue damage. We have previously shown that the perfusion of donor hearts with CM protects against myocardial heart transplantation-induced ischemia/reperfusion injury in 15-month-old rats [37]. Additionally, we have reported that the preservation of brain-dead donor hearts with cardioplegic solution-enriched CM improves cardiac graft contractility after transplantation [31]. It should be noted the CM has no protective effect on IR-induced impaired contractile response in nondiabetic aortic rings. We can speculate that CM has a much larger positive impact in diabetic aortic rings than healthy ones, because contraction to PE and caspase-3, caspase-8, caspase-9, and caspase-12 immunoreactivity were severely impaired in the DM-IR compared to control-IR rats. Taking this into consideration, the expected effect of CM treatment may be higher in the DM-IR group.

Some limitations need to be acknowledged. First, aortic function was studied ex vivo, and the involvement of nonaortic tissues, blood flow to these tissues, and leukocyte activation need to be translated into a clinically relevant *in vivo* situation. Second, further studies are required to confirm the effects of IR injury on human internal mammary arteries and saphenous veins from diabetic patients undergoing CABG. Third, even though we showed that targeting caspase-3, caspase-8, caspase-9, and caspase-12 may partially be responsible for the protective effect of CM on impaired endothelium-dependent and endothelium-independent smooth muscle relaxation of vascular grafts from diabetic rats, their activation was not assessed. Lastly, other specific pathways involved in diabetic vascular complications were not investigated.

## 5. Conclusions

Our results provide novel experimental evidence that the preservation of vascular grafts with CM improves impaired endothelial function, smooth muscle relaxation, and contraction following *in vitro* IR injury in diabetic rats. This protective effect may be, in part, related to a mitigated increase in caspase-3, caspase-8, caspase-9, and caspase-12 and decrease in apoptotic DNA breakage.

## Figures and Tables

**(a):** 

	Control	DM
Body weight (g)	575 ± 16	348 ± 25^∗^
Intima-media thickness to body weight (mm/g) (×1,000)	0.23 ± 0.01	0.34 ± 0.02^∗^
Intima-media area to body weight (mm^2^/g) (×1,000)	2.42 ± 0.08	3.84 ± 0.22^∗^
Lumen area to body weight (mm^2^/g) (×1,000)	6.53 ± 0.29	11.31 ± 0.62^∗^
Intima-media/lumen area ratio	0.373 ± 0.031	0.340 ± 0.007^∗^

**(b):** 

	Control (**n** = 31-32 rings, 8 rats)	Control-IR (**n** = 30-31 rings, 8 rats)	DM (**n** = 31-32 rings, 8 rats)	DM-IR (**n** = 40-44 rings, 10-11 rats)
PE (g)	2.90 ± 0.15	2.20 ± 0.15^∗^	3.00 ± 0.17	3.25 ± 0.19^#^
PE (% of KCl)	55.5 ± 2.7	80.3 ± 5.5^∗^	78.4 ± 4.0^∗^	168.3 ± 11.5^∗,#,$^
pD_2_ to PE	6.97 ± 0.04	6.94 ± 0.11	7.04 ± 0.04	7.68 ± 0.11^#,$^
KCl (g)	5.20 ± 0.11	2.91 ± 0.17^∗^	3.90 ± 0.12^∗^	1.88 ± 0.09^#,$^
*R*_max_ to ACh (%)	88.2 ± 0.9	56.3 ± 2.7^∗^	86.7 ± 0.1	42.5 ± 2.5^$^
pD_2_ to ACh	7.54 ± 0.06	7.14 ± 0.24	7.43 ± 0.06	6.03 ± 0.22^#,$^
*R*_max_ to SNP (%)	99.5 ± 0.3	97.7 ± 0.4^∗^	99.1 ± 0.4	93.2 ± 1.4^$^
pD_2_ to SNP	9.30 ± 0.03	9.13 ± 0.05^∗^	9.51 ± 0.1	8.93 ± 0.07^$^

Data are represented as the mean ± SEM. DM: diabetes mellitus; IR: ischemia/reperfusion; PE: phenylephrine; KCl: potassium chloride; ACh: acetylcholine; SNP: sodium nitroprusside; *R*_max_: maximum relaxation; pD_2_: negative logarithm of the corresponding half-maximum response (EC_50_). ^∗^*p* < 0.05 versus control; ^#^*p* < 0.05 versus control-IR; ^$^*p* < 0.05 versus DM.

**(a):** 

	Control (**n** = 31-32, 8 rats)	Control-IR (**n** = 30-31, 8 rats)	Control-IR+CM (**n** = 40, 10 rats)
PE (g)	2.90 ± 0.15	2.20 ± 0.15^∗^	2.74 ± 0.12^#^
PE (% of KCl)	55.5 ± 2.7	80.3 ± 5.5^∗^	86.1 ± 3.8^∗^
pD_2_ to PE	6.97 ± 0.04	6.94 ± 0.11	6.87 ± 0.11
KCl (g)	5.20 ± 0.11	2.91 ± 0.17^∗^	3.36 ± 0.16^∗^
*R*_max_ to ACh (%) (5x10^−6^M)	88 ± 1	56 ± 3^∗^	65 ± 2^∗,#^
pD_2_ to ACh	7.54 ± 0.06	7.14 ± 0.24	7.42 ± 0.09
*R*_max_ to SNP (%)(5x10^−7^M)	99.5 ± 0.3	97.7 ± 0.4	98.0 ± 0.4
pD_2_ to SNP	9.30 ± 0.03	9.13 ± 0.05^∗^	9.21 ± 0.04^∗^

**(b):** 

	DM (**n** = 31-32, 8 rats)	DM-IR (**n** = 40-44, 10-11 rats)	DM-IR+CM (**n** = 43-44, 11 rats)
PE (g)	3.00 ± 0.16	3.25 ± 0.19	3.15 ± 0.12
PE (% of KCl)	78.4 ± 4.0	168.3 ± 11.5^∗^	141.6 ± 8.3^∗^
pD_2_ to PE	7.04 ± 0.04	7.68 ± 0.11^∗^	7.20 ± 0.05^#^
KCl (g)	3.90 ± 0.12	1.88 ± 0.09^∗^	2.34 ± 0.10^∗^
*R*_max_ to ACh (%)	86.7 ± 0.1	42.5 ± 2.5^∗^	61.9 ± 2.0^∗,#^
pD_2_ to ACh	7.43 ± 0.06	6.03 ± 0.22^∗^	7.12 ± 0.07^∗,#^
*R*_max_ to SNP (%)(5x10^−7^M)	99.1 ± 0.4	93.2 ± 1.4^∗^	98.7 ± 0.4^#^
pD_2_ to SNP	9.51 ± 0.09	8.93 ± 0.07^∗^	9.11 ± 0.03^∗^

Data are represented as the mean ± SEM. PE: phenylephrine; KCl: potassium chloride; ACh: acetylcholine; SNP: sodium nitroprusside; *R*_max_: maximum relation; pD_2_: negative logarithm of the corresponding half-maximum response (EC_50_). Data were represented as mean SEM. ^∗^*p* < 0.05 versus corresponding control or DM; ^#^*p* < 0.05 versus corresponding control-IR or DM-IR.

## Data Availability

Data associated with this study is available upon reasonable request.

## Supplementary Materials

Supplementary Materials Figure S1: characterization of aortic histomorphometry diabetic rats. Representative hematoxylin and eosin-stained sections of nondiabetic control (left panel) and diabetic (right panel) thoracic aortas. (A) shows ×4 magnification bar: 500 *μ*m; and (B) shows ×20 magnification bar: 100 *μ*m. DM indicated diabetes mellitus. Table S1: list of factors present in bone marrow culture-derived conditioned medium (CM). We previously reported the relative expression of proteins in our CM by rat cytokine antibody array coated with 90 antibodies (BioCat GmbH, Heidelberg, Germany) [14]. This array showed that CM contains 23 proteins involved in either apoptosis, inflammation, or oxidative stress [14].

## References

[1] Neumann F. J., Hochholzer W., Siepe M. (2018). ESC/EACTS guidelines on myocardial revascularization 2018: the most important innovations. *Herz*.

[2] Eefting F., Rensing B., Wigman J. (2004). Role of apoptosis in reperfusion injury. *Cardiovascular Research*.

[3] Orasanu G., Plutzky J. (2009). The pathologic continuum of diabetic vascular disease. *Journal of the American College of Cardiology*.

[4] Beckman J. A., Creager M. A., Libby P. (2002). Diabetes and atherosclerosis. *JAMA*.

[5] Rahman S., Rahman T., Ismail A. A., Rashid A. R. (2007). Diabetes-associated macrovasculopathy: pathophysiology and pathogenesis. *Diabetes, Obesity & Metabolism*.

[6] Thourani V. H., Weintraub W. S., Stein B. (1999). Influence of diabetes mellitus on early and late outcome after coronary artery bypass grafting. *The Annals of Thoracic Surgery*.

[7] Price M. J., Chou C. C., Frantzen M. (2006). Intravenous mesenchymal stem cell therapy early after reperfused acute myocardial infarction improves left ventricular function and alters electrophysiologic properties. *International Journal of Cardiology*.

[8] Shake J. G., Gruber P. J., Baumgartner W. A. (2002). Mesenchymal stem cell implantation in a swine myocardial infarct model: engraftment and functional effects. *The Annals of Thoracic Surgery*.

[9] Barbash I. M., Chouraqui P., Baron J. (2003). Systemic delivery of bone marrow-derived mesenchymal stem cells to the infarcted myocardium. *Circulation*.

[10] Gnecchi M., He H., Liang O. D. (2005). Paracrine action accounts for marked protection of ischemic heart by Akt- modified mesenchymal stem cells. *Nature Medicine*.

[11] Kinnaird T., Stabile E., Burnett M. S. (2004). Local delivery of marrow-derived stromal cells augments collateral perfusion through paracrine mechanisms. *Circulation*.

[12] Caplan A. I., Dennis J. E. (2006). Mesenchymal stem cells as trophic mediators. *Journal of Cellular Biochemistry*.

[13] Timmers L., Lim S. K., Arslan F. (2007). Reduction of myocardial infarct size by human mesenchymal stem cell conditioned medium. *Stem Cell Research*.

[14] Korkmaz-Icoz S., Zhou P., Guo Y. (2021). Mesenchymal stem cell-derived conditioned medium protects vascular grafts of brain-dead rats against in vitro ischemia/reperfusion injury. *Stem Cell Research & Therapy*.

[15] Korkmaz-Icoz S., Sun X., Li S. (2021). Conditioned medium from mesenchymal stem cells alleviates endothelial dysfunction of vascular grafts submitted to ischemia/reperfusion injury in 15-month-old rats. *Cell*.

[16] Radovits T., Korkmaz S., Loganathan S. (2009). Comparative investigation of the left ventricular pressure-volume relationship in rat models of type 1 and type 2 diabetes mellitus. *American Journal of Physiology. Heart and Circulatory Physiology*.

[17] Veres G., Hegedűs P., Barnucz E. (2013). Addition of vardenafil into storage solution protects the endothelium in a hypoxia-reoxygenation model. *European Journal of Vascular and Endovascular Surgery*.

[18] Korkmaz-Icoz S., Vater A., Li S. (2015). Mild type 2 diabetes mellitus improves remote endothelial dysfunction after acute myocardial infarction. *Journal of Diabetes and its Complications*.

[19] Korkmaz-Icoz S., Brlecic P., Ruppert M., Radovits T., Karck M., Szabó G. (2018). Mechanical pressure unloading therapy reverses thoracic aortic structural and functional changes in a hypertensive rat model. *Journal of Hypertension*.

[20] Dileepan K. N., Johnston T. P., Li Y., Tawfik O., Stechschulte D. J. (2004). Deranged aortic intima-media thickness, plasma triglycerides and granulopoiesis in Sl/Sl(d) mice. *Mediators of Inflammation*.

[21] Li S., Korkmaz S., Loganathan S. (2012). Acute ethanol exposure increases the susceptibility of the donor hearts to ischemia/reperfusion injury after transplantation in rats. *PLoS One*.

[22] Li S., Loganathan S., Korkmaz S. (2015). Transplantation of donor hearts after circulatory or brain death in a rat model. *The Journal of Surgical Research*.

[23] Beckmann A., Meyer R., Lewandowski J., Markewitz A., Harringer W. (2019). German heart surgery report 2018: the Annual Updated Registry of the German Society for Thoracic and Cardiovascular Surgery. *The Thoracic and Cardiovascular Surgeon*.

[24] Furchgott R. F., Vanhoutte P. M. (1989). Endothelium-derived relaxing and contracting factors. *The FASEB Journal*.

[25] Ignarro L. J., Buga G. M., Wood K. S., Byrns R. E., Chaudhuri G. (1987). Endothelium-derived relaxing factor produced and released from artery and vein is nitric oxide. *Proceedings of the National Academy of Sciences of the United States of America*.

[26] Moncada S., Gryglewski R., Bunting S., Vane J. R. (1976). An enzyme isolated from arteries transforms prostaglandin endoperoxides to an unstable substance that inhibits platelet aggregation. *Nature*.

[27] Griffith T. M. (2004). Endothelium-dependent smooth muscle hyperpolarization: do gap junctions provide a unifying hypothesis?. *British Journal of Pharmacology*.

[28] Lejay A., Fang F., John R. (2016). Ischemia reperfusion injury, ischemic conditioning and diabetes mellitus. *Journal of Molecular and Cellular Cardiology*.

[29] Elmore S. (2007). Apoptosis: a review of programmed cell death. *Toxicologic Pathology*.

[30] Li S. Y., Qi Y., Hu S. H. (2017). Mesenchymal stem cells-conditioned medium protects PC12 cells against 2,5-hexanedione-induced apoptosis via inhibiting mitochondria-dependent caspase 3 pathway. *Toxicology and Industrial Health*.

[31] Korkmaz-Icoz S., Li K., Loganathan S. (2020). Brain-dead donor heart conservation with a preservation solution supplemented by a conditioned medium from mesenchymal stem cells improves graft contractility after transplantation. *American Journal of Transplantation*.

[32] Bond M., Murphy G., Bennett M. R., Newby A. C., Baker A. H. (2002). Tissue inhibitor of metalloproteinase-3 induces a Fas-associated death domain- dependent type II apoptotic pathway. *The Journal of Biological Chemistry*.

[33] Gerber H. P., McMurtrey A., Kowalski J. (1998). Vascular endothelial growth factor regulates endothelial cell survival through the phosphatidylinositol 3′-kinase/Akt signal transduction pathway:. *The Journal of Biological Chemistry*.

[34] DeGracia D. J., Montie H. L. (2004). Cerebral ischemia and the unfolded protein response. *Journal of Neurochemistry*.

[35] Lenna S., Han R., Trojanowska M. (2014). Endoplasmic reticulum stress and endothelial dysfunction. *IUBMB Life*.

[36] Xue L. X., Liu H. Y., Cui Y. (2017). Neuroprotective effects of Activin A on endoplasmic reticulum stress-mediated apoptotic and autophagic PC12 cell death. *Neural Regeneration Research*.

[37] Korkmaz-Icoz S., Li S., Huttner R. (2019). Hypothermic perfusion of donor heart with a preservation solution supplemented by mesenchymal stem cells. *The Journal of Heart and Lung Transplantation*.

[38] Mondello C., Scovassi A. I. (2010). Apoptosis: a way to maintain healthy individuals. *Sub-Cellular Biochemistry*.

