# Supplementary material for: Bone Marrow Culture-Derived Conditioned Medium Recovers Endothelial Function of Vascular Grafts following In Vitro Ischemia/Reperfusion Injury in Diabetic Rats
Source: Stem Cells Int. 2022 Oct 14;2022:7019088. doi: 10.1155/2022/7019088 (PMC9586819; doi:10.1155/2022/7019088)
Supplement: Supplementary Materials — Figure S1: characterization of aortic histomorphometry diabetic rats. Representative hematoxylin and eosin-stained sections of nondiabetic control (left panel) and diabetic (right panel) thoracic aortas. (A) shows ×4 magnification bar: 500 μm; and (B) shows ×20 magnification bar: 100 μm. DM indicated diabetes mellitus. Table S1: list of factors present in bone marrow culture-derived conditioned medium (CM). We previously reported the relative expression of proteins in our CM by rat cytokine antibody array coated with 90 antibodies (BioCat GmbH, Heidelberg, Germany) [14]. This array showed that CM contains 23 proteins involved in either apoptosis, inflammation, or oxidative stress [14]. [file 7019088.f1.zip › Online Table 1_Factors in CM_Stem Cells Int R2.docx]

**Online Table S1. List of factors present in bone marrow culture-derived conditioned medium (CM).** We previously reported the relative expression of proteins in our CM by rat cytokine antibody array coated with 90 antibodies (BioCat GmbH, Heidelberg, Germany) [1]. This array showed that CM contains 23 proteins involved in either apoptosis, inflammation, or oxidative stress [1].

| **Phenomenon** | **Properties** | **Factors** |
| --- | --- | --- |
| Apoptosis | Anti-apoptotic | TIMP-1, Growth Hormone, Growth Hormone Receptor, EG-VEGF (PK1), VEGF, VEGF-C, Activin A, BDNF, FGF-BP |
|  | Pro-apoptotic | TRAIL, Thrombospondin, TROY, Fas Ligand/TNFSF6 |
| Inflammation | Anti-inflammatory | CXCR-4, MDC |
|  | Pro-inflammatory | CINC-2, CINC-3, FSL-1, MCP-1, MDC, MIF, MIP-1α, MMP-13, TLR4, Fas Ligand/TNFSF6, CXCR-4 |
| Oxidative stress | Protection | FGF-BP |

TIMP-1 indicates tissue inhibitors of metalloproteinases-1; EG-VEGF, endocrine gland-derived vascular endothelial growth factor; PK1, prokineticin 1; VEGF, vascular endothelial growth factor; BDNF, brain-derived neurotrophic factor; FGF-BP, fibroblast growth factor-binding protein; TRAIL, tumor necrosis factor-related apoptosis-inducing ligand; TROY, tumor necrosis factor receptor superfamily, member 19; TNFSF6, tumor necrosis factor superfamily, member 6; CXCR-4, C-X-C chemokine receptor type 4; MDC, macrophage derived chemokine; CINC, cytokine-induced neutrophil chemoattractant; FSL-1, follostatin-like-1; MCP-1, monocyte chemoattractant protein-1; MIF, macrophage migration inhibitory factor; MIP-1α, macrophage inflammatory proteins 1 alpha; MMP-13, matrix metalloproteinase-13; TLR4, toll like receptor 4*.*

**Reference**

[1] Korkmaz-Icoz, S., Zhou, P., Guo, Y., et al., "Mesenchymal stem cell-derived conditioned medium protects vascular grafts of brain-dead rats against in vitro ischemia/reperfusion injury," *Stem Cell Res Ther,* vol. 12***,*** no. 1, pp. 144, 2021.
