# Supplementary figures and images for: Bone Marrow Culture-Derived Conditioned Medium Recovers Endothelial Function of Vascular Grafts following In Vitro Ischemia/Reperfusion Injury in Diabetic Rats
Source: Stem Cells Int. 2022 Oct 14;2022:7019088. doi: 10.1155/2022/7019088 (PMC9586819; doi:10.1155/2022/7019088)

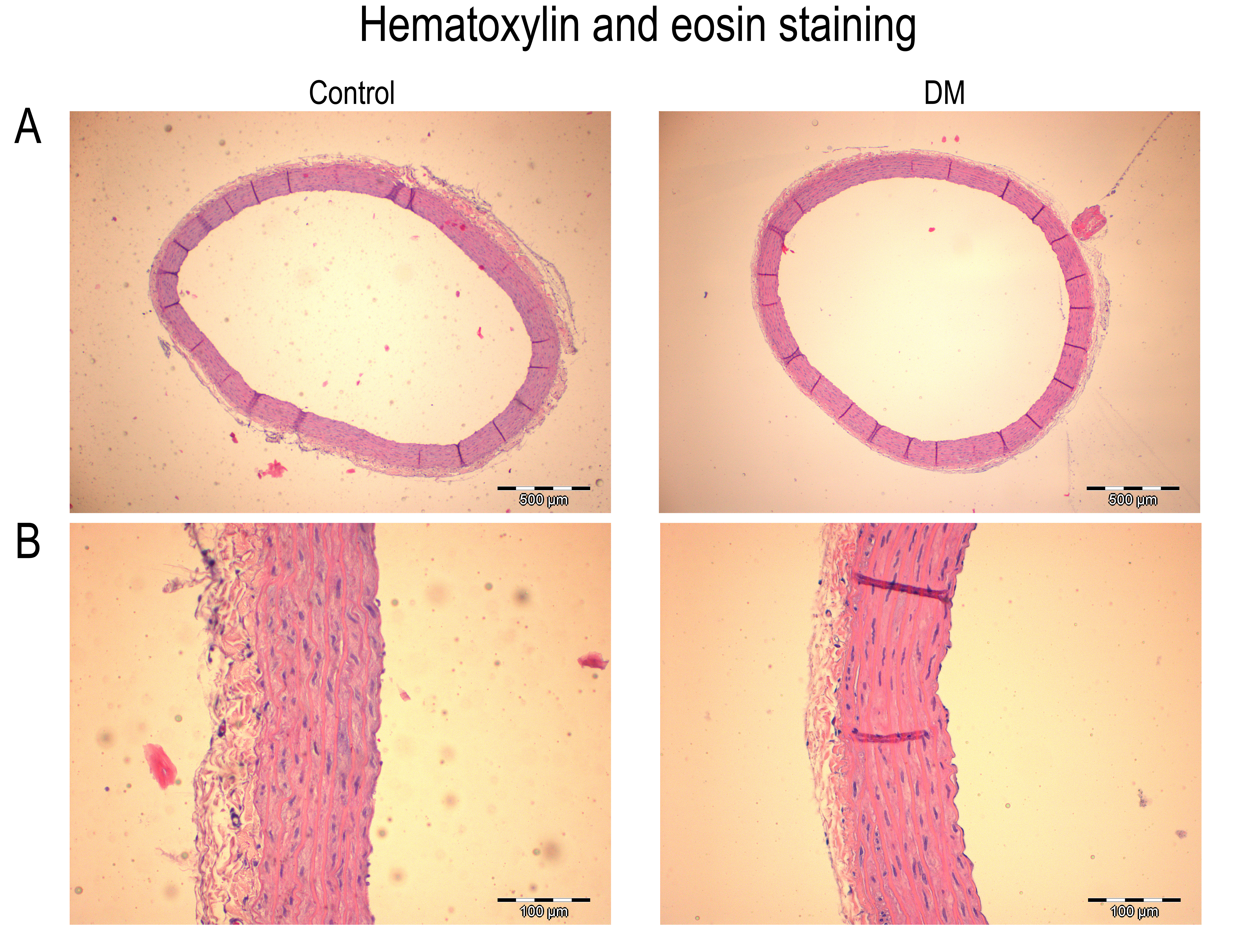

Supplement: Supplementary Materials — Figure S1: characterization of aortic histomorphometry diabetic rats. Representative hematoxylin and eosin-stained sections of nondiabetic control (left panel) and diabetic (right panel) thoracic aortas. (A) shows ×4 magnification bar: 500 μm; and (B) shows ×20 magnification bar: 100 μm. DM indicated diabetes mellitus. Table S1: list of factors present in bone marrow culture-derived conditioned medium (CM). We previously reported the relative expression of proteins in our CM by rat cytokine antibody array coated with 90 antibodies (BioCat GmbH, Heidelberg, Germany) [14]. This array showed that CM contains 23 proteins involved in either apoptosis, inflammation, or oxidative stress [14]. [file 7019088.f1.zip › Online Fig1_HE_Stem Cells Int (1).jpg]
